# Evidence for predilection of macrophage infiltration patterns in the deeper midline and mesial temporal structures of the brain uniquely in patients with HIV-associated dementia

**DOI:** 10.1186/1471-2334-9-192

**Published:** 2009-12-02

**Authors:** Li Zhou, Rejane Rua, Thomas Ng, Valentina Vongrad, Yung S Ho, Carolyn Geczy, Kenneth Hsu, Bruce J Brew, Nitin K Saksena

**Affiliations:** 1 Retroviral Genetics Division, Center for Virus Research, Westmead Millennium Institute, Westmead Hospital, The University of Sydney, Westmead, NSW 2145, Sydney, Australia; 2 Department of Anatomical Pathology, ICPMR, Westmead Hospital, Westmead, NSW 2145. Sydney, Australia; 3 Inflammatory Diseases Research Unit, School of Medical Sciences, University of NSW, Sydney, NSW, 2052, Australia; 4 Department of Neurology, St. Vincent's Hospital, Darlinghurst, Sydney, Australia; 5 Ecole Normale Superieure, 45 Rue Ulm, 75005 Paris, France

## Abstract

**Background:**

HIV-1 penetrates the central nervous system, which is vital for HIV-associated dementia (HAD). But the role of cellular infiltration and activation together with HIV in the development of HAD is poorly understood.

**Methods:**

To study activation and infiltration patterns of macrophages, CD8+ T cells in relation to HIV in diverse CNS areas of patients with and without dementia. 46 brain regions from two rapidly progressing severely demented patients and 53 regions from 4 HIV+ non-dementia patients were analyzed. Macrophage and CD8+ T cell infiltration of the CNS in relation to HIV was assessed using immuno-histochemical analysis with anti-HIV (P24), anti-CD8 and anti-CD68, anti-S-100A8 and granzyme B antibodies (cellular activation). Statistical analysis was performed with SPSS 12.0 with Student's t test and ANOVA.

**Results:**

Overall, the patterns of infiltration of macrophages and CD8+ T cells were indiscernible between patients with and without dementia, but the co-localization of macrophages and CD8+ T cells along with HIV P24 antigen in the deeper midline and mesial temporal structures of the brain segregated the two groups. This predilection of infected macrophages and CD8+ T cells to the middle part of the brain was unique to both HAD patients, along with unique nature of provirus gag gene sequences derived from macrophages in the midline and mesial temporal structures.

**Conclusion:**

Strong predilection of infected macrophages and CD8+ T cells was typical of the deeper midline and mesial temporal structures uniquely in HAD patients, which has some influence on neurocognitive impairment during HIV infection.

## Background

Human immunodeficiency virus type 1 (HIV-1) is associated with the development of neurological complications in many infected individuals, most especially a broad spectrum of motor impairments and cognitive deficits. Approximately 80-90% of autopsied cases of HIV-1-infected people demonstrated neuropathological changes [1-4]. The histopathology of HIV-associated dementia (HAD) is characterized by brain infiltration of mononuclear cells, formation of multinucleated giant cells, astrogliosis, and neuronal damage sometimes with neuronal loss [5,6]. The underlying mechanisms of HAD leading to neurological disorders and its complete understanding is still lacking. In addition, after the introduction of highly active antiretroviral therapy (HAART), the prevalence of HAD has risen due to prolonged life expectancy of HIV-infected patients [7-9].

HIV-1 penetration of the central nervous system is a vital event in the neuropathogenesis of HAD. The presence of HIV in the cerebrospinal fluid (CSF) is one of the factors implicated in HAD [10-12], although high plasma viral load do not necessarily correlate with dementia. The principal cell types infected by HIV in the CNS and implicated in HIV related neuronal dysfunction are macrophages and microglia, which are known to secrete cytokines and factors toxic to neurons [13]. It is also widely believed that monocytes or monocyte-derived macrophages may be required for neurologic manifestation of HIV disease [14,15]. Blood-borne macrophages can transmit the virus into the CNS and then infect or stimulate other perivascular macrophages and microglia [12,16]. However, HAD usually occurs at an advanced stage of HIV disease, while HIV entry into the CNS has been reported to occur early after primary infection [17,18]. The most popular explanation for this discrepancy is the collapse of immune functions mediated by T cells because cytotoxic T lymphocytes, which are believed to be the principal regulatory elements that control viral production in the periphery and CNS [19-23]. Both CD4+ and CD8+ T lymphocytes have been shown to accumulate in AIDS patients with HIV encephalitis along with the demonstration that brain CD8-CTL are HIV-specific and are associated with HIV encephalitis [24-27].

Although some studies have shown evidence in favor of frequency and topographical distribution of HIV core protein P24 [28,29], detailed investigations with focus on quantity, quality, topographical distribution and infiltration of macrophages, CD8+ T cells, especially in relation to HIV, in diverse regions of the brain from patients with and without dementia, which might elucidate entry mechanism of HIV into the CNS and explain regional involvement in the development of HAD, are seriously lacking. Therefore, we have carried out a detailed and simultaneous tracking of activation and infiltration patterns of macrophages, CD8+ T cells in relation to HIV P24 antigen in diverse areas of the brain of HIV+ patients with and without dementia. We analyzed 53 different brain regions from 4 HIV+ non-dementia patients and 46 regions from patients with 2 HIV+ severely demented, rapidly progressing patients. Our study is novel in revealing the predilection of HIV movements together with cellular infiltrates of macrophages and CD8+ T cells to the deeper mid-line and mesial structures uniquely in patients with HAD.

## Methods

### Brain tissue collection and ethical issues

Brain samples were obtained from the Westmead Hospital, Sydney, Australia (Reference No: 5465). Clinical profiles of all patients are shown in Table 1. The autopsies were selected from a variety of brain regions of 2 patients with severe dementia and 4 without dementia. 46 different brain regions from patients with dementia and 53 from patients without dementia were used for this study (Table 2). Use of samples in this study was approved by the Institutional Review Board and the Ethics Committee of the University of Sydney and The Westmead Hospital individually. The family members of the patients have given written, informed consent. For the diagnostic criteria for HAD, the American Academy of Neurology 1991 criteria were used (*American Academy of Neurology. Nomenclature and research case definitions for neurological manifestations of HIV type 1 infection 1: Report of a working group of AAN of neurology and AIDS task force. 1991*).

**Table 1:** Patient clinical details

patient	sex^a^	Age at death	ADC stage^b^	Viral load/ml plasma	Viral load/ml CSF	ART	Non-HIVE neuropathology	Duration from seropositive for HIV to death(year)	Postmortem interval (h)
A	F	44	L	2.96 × 10^5^	7.6 × 10^5^	AZT, DDI		6	15
B	M	3	L	3.6 × 10^5^	1 × 10^6^	AZT, DDI,		3/4	No record
C	M	48	N	8.2 × 10^5^	2 × 10^3^	No record	CMV ventriculitis and encephalitis	1/2	20
D	M	41	N	2.9 × 10^5^	3.3 × 10^4^	No record	Cerebral B cell lymphoma	No record	53
E	M	64	N	2 × 10^4^	1 × 10^3^	AZT, Mycelex, Clotrimazole	Malignant lymphoma	6	21
F	M	44	N	N/A	N/A		Spongiform myelopathy of spinal cord	7	46.5

^a ^M, male; F, female

^b ^L, late stage of dementia; N, without dementia; N/A = not available

**Table 2:** Patient tissue samples available, which were used in the study

patient tissue sample^a^	A	B	C	D	E	F
Left Frontal lobe	A	A	A		A	
Right Frontal lobe	A	A	A	A		
Left Parietal lobe	A	A	A		A	
Right Parietal lobe	A	A	A	A		
Left Temporal lobe	A				A	
Right Temporal lobe	A					
Left Occipital lobe	A	A	A		A	A
Right Occipital lobe	A	A	A	A		
Left Basal ganglia	A	A	A			A
Right Basal ganglia	A	A	A	A		
Left Hippocampus	A	A	A			A
Right Hippocampus	A		A	A		
Left Cerebellum	A	A	A			A
Right Cerebellum	A	A	A	A		
Mid brain	A	A	A	A		A
Pons	A	A	A	A		A
Upper medulla	A	A	A	A		A
Lower medulla	A					
Left thalamus	A		A	A		
Right thalamus	A	A	A			
Corpus callosum		A	A			A
Pituitary		A				
Mamlliary bodies		A				A
Choroid plexus					A	
putamen					A	

^a^A, availability of tissue specimen; empty block, no tissue specimen available.

### Histology and immunohistochemistry

Tissues were processed, and stained histologically and immunohistochemically as previously described[30]. Immunofluorosecent double staining was performed using http://www.ihcworld.com/_protocols/general_IHC/immunofl_double_squential.htm the online protocol. Antibodies CD8 and CD68 (Ventana) were used to detect lymphocytes and macrophages, anti-granzyme B antibody was used to detect CD8 CTL, whereas P24 (1:10, DAKO) was used to detect HIV-infected cells. S100A8 was a gift from Dr. Caroline Geczy and it was employed together with the anti-CD68 to identify the activated macrophages, since it recognizes Calgranulin A, a S100 calcium-binding protein MRP-8 (S100A8) which is only expressed in chronically activated macrophages. HIV positive and negative inflamed tonsils were used as positive controls for anti-P24 and anti-CD8 and CD68, respectively, while the deletion of primary antibodies served as negative controls (see additional file 1). The numbers of the positive cells were manually counted. All the slides were viewed at magnification × 200. Grading standards used for validating histopathological studies: Severe microglia nodule: 4 or more than 4 nodules observed in average of 10 microscopic fields. Extensive astrocytosis: >30 activated astrocytes were observed in an average of 1 microscopic field. Severe encephalopathy: >7/10 fields showing pathological change consistent with encephalopathy.

### Laser-capture microscopy (LCM)

HIV-infected macrophages were identified through double staining with anti-P24/CD68 antibodies. Individual cells were catapulted and captured with a 2-millisecond shot of a 7.5 μm infrared diode laser beam set to 60 mV, onto flat top 0.2 μl PCR tube using a PALM MicroBeam system (P.A.L.M. Mikrolaser Technologie, Bernried, Germany). Tissue (6 um thickness) obtained from an average of 1500 firings was captured.

### PCR, cloning, sequence and phylogenetic analysis

The tissue sampled by LCM was heated at 95°c for 30 mins and was used in the nested PCR reaction. A 325 bp gag-pol gene fragment was amplified. The PCR, cloning, sequencing and phylogenetic analyses of 325 bp fragment was performed as previously reported [31]. Both phylogenetic estimations were also repeated using bootstrap simulation of 2000 repetitions with replacements. The same sequence alignment were ran in SplitsTree version 4 [32] using Median Network [33] with a convex hull splits representation. The pol amino acid alignment was re-analyzed with Neighbor joining and Parsimony estimations. The resulting phylogeny was analyzed in MacClade Version 4.08 to trace the character changes in the alignment that were consistent in the evolutionary tree predicted.

### Statistical analysis

Data were log-transformed and analyzed by t-tests and rank correlation. General linear models with patient ID as a random effect and location as a fixed factor were used to assess the effect of location within patients. The software package SPSS 12.0 for Windows was used to analyze the data. Two-tailed tests with a significance level of 5% were used throughout.

## Results

### Histopathology and immunohistochemistry

#### Region-specific HIV pathology and cellular-infiltration of macrophages and CD8+ T cells in relation to HIV in the deeper midline and mesial temporal structures is unique to patients with HAD

**Figure 1:**
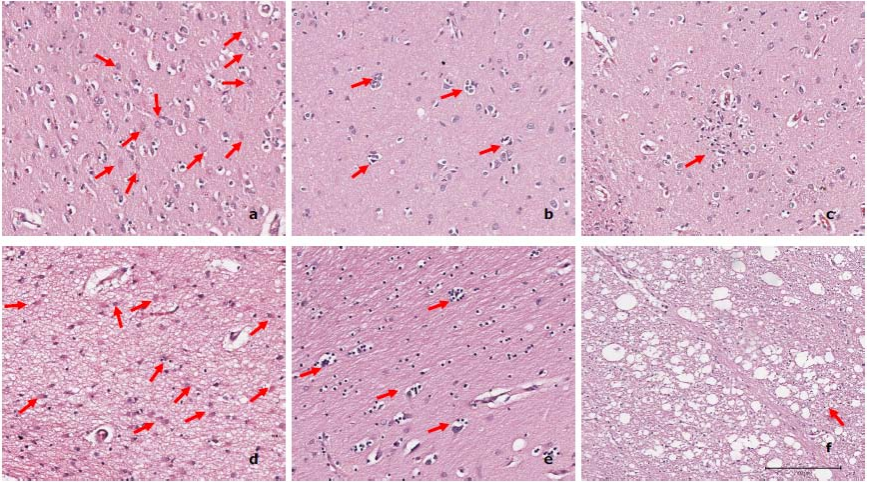
**Histopathology of different brain regions of HAD patient A (a-c) and patient B (d-f)**. Extensive astrocytosis (a and d) was seen in most brain regions in case of both HAD patients (13/26 in the patient A and 11/20 in the patient B). Focal neurophagia was observed in the left occipital lobe of patient A (b) and left hippocampus of patient B (e) individually, although it was only found in very limited regions (4/26 in the patient A and 1/20 in the patient B). Severe microglial nodules (c) were been observed in 12/26 regions of patient A, but the absence in the patient B. Encephalopathy (f) was identified in 5/26 regions in the patient A and 3/20 in the patient B, respectively. Arrows in the figure signify each of the pathologic features outlined above. **a**: right basal ganglia, **b**: left occipital lobe, **c**: left basal ganglia, **d**: left basal ganglia, **e**: left hippocampus, **f**: mid brain.

In the 2 rare rapidly progressing dementia patients, the histopathological examination showed severe histopathology typical of HAD[5,6] in most regions (Fig.1). In contrast, in HIV non-dementia patients, we failed to find similar or even comparable degree of pathological changes to HAD patients. Notably, regional pathological variations were observed in 2 HAD patients in the deeper midline and mesial temporal structures, which were more involved pathologically as compared to the cerebral lobes. Further, neuronophagia observed was highly focal in the left occipital lobe in case of patient A and left hippocampus for patient B (Fig.1b and 1e). Between two dementia patients, the pathological changes in the patient A were much more severe than in the patient B, which was consistent with severe microglial nodulation (Fig. 1c) observed in the left temporal lobe, left basal ganglia, left hippocampus, left cerebellum, mid brain, pons, upper medulla, lower medulla, right frontal lobe, right temporal lobe, right occipital lobe, and right basal ganglia, respectively, in patient A and none in patient B.

**Figure 2:**
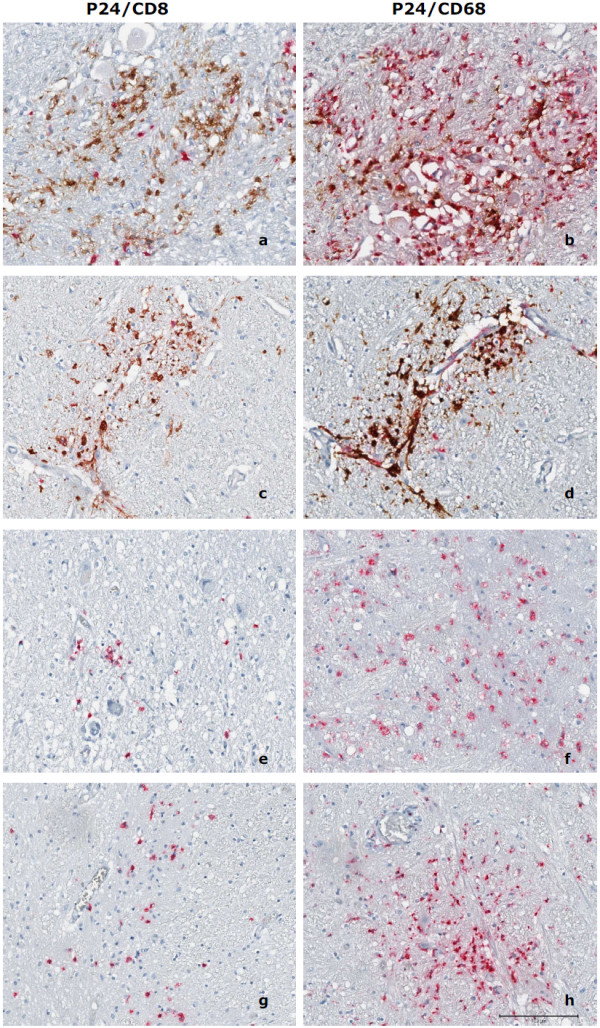
**Immunohistochemistry P24/CD8 and P24/CD68 double staining of HAD and HIV non-dementia patients**. Immunohistochemistry P24(brown)/CD8(red) and P24(brown)/CD68(red) double staining of HAD (patient A: a and b, patient B: c and d) and HIV non-dementia (patient c: e and f, patient d: g and h) patients in medulla region. Extensive P24 staining was observed in HAD patients (a, b, c and d) but was entirely absent in HIV non-dementia patients (e, f, g and h). It was also noticed that CD68+ macrophages co-localized with P24 (b and d) within HAD patients, whereas the co-localization of CD8 and P24 is much less pronounced (a and c). Most infected cells were macrophages (b and d), although there were still few CD8+ T cells infected as well. We observed noticeable CD8 (e and g) and CD68 (f and h) positive cells in HIV non-dementia patients, especially around the perivascular area, but these cells were negative for P24 antigen in all the brain regions studied for all 4 HIV non-dementia patients.

By immunohistochemistry, we first assessed the infiltration of CD68+ macrophages and CD8+ T cells individually, followed by double staining with anti-P24 antibody for topographical tracking and localization of these two cell types in relation to HIV in diverse areas of the CNS from patients with and without dementia. Extensive and diffused presence of CD68+ cells and very mild and focal CD8+ T cell infiltration was found in the HAD brain. However, based on cellular infiltration of CD68+ and CD8+ cells alone (not taking HIV into account), we could not distinguish between patients with and without dementia (Fig.2). Importantly, the P24 staining truly segregated patients with and without dementia, demonstrating the complete absence of P24 positive cells in 53/53 regions analyzed from all 4 HIV non-dementia patients (Fig.2e, f, g and 2h, **and see additional file **2**for more brain regions**). In contrast, both HAD patients showed the majority of CNS regions positive for P24 staining (Fig.2a, b, c and 2d). We observed a strong positive relationship between macrophages and/or CD8 and P24 positive cells by rank correlation analysis (p < 0.05), although the co-localization of CD68+ cells with P24+ cells was much more pronounced (Fig.2b and 2d), whereas only to a lesser degree were CD8+ T cells and P24 antigen+ cells were seen to be co-localizing in these two severe HAD patients (Fig.2a and 2c).

#### Similar distributional trends of CD68+ macrophage and CD8+ T cell infiltration in relation to HIV in patients with and without dementia: statistical validation

**Figure 3:**
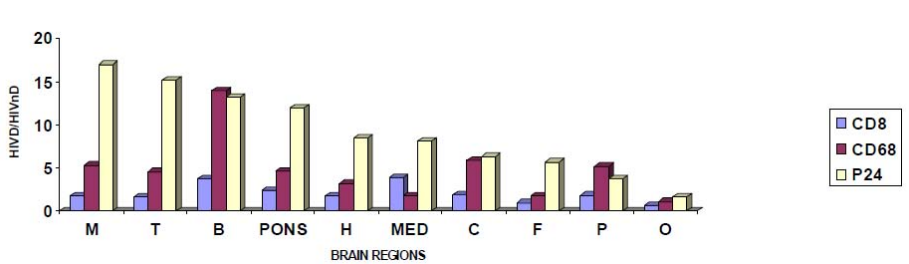
**Ratio of CD8, CD68 and P24 between HAD and HIV non-dementia patients**. The mean value of the log-transformed CD8, CD68 and P24 data for each patient in each region was calculated and analyzed with two-tailed tests with a significance level of 5% (Because several zero counts were observed, one was added to the observed count prior to log transformation). In order to show the difference of value in different regions, only the ratio of mean value has been shown between HAD and HIV non-dementia patients. For CD8, there was significant difference between HAD and HIV non-dementia patients in the basal ganglia and parietal regions (P < 0.05), for CD68, the difference was observed in basal ganglia, parietal and mid brain (P < 0.05), whereas for P24 antigen, every region showed statistically significant difference, which is exemplified by the absence of P24 antigen in all HIV non-dementia patients (P < 0.05). M: mid brain, T: temporal lobe, B: basal ganglia, H: hippocampus, MED: medulla, C: cerebellum, F: frontal lobe, P: parietal lobe, O: occipital lobe.

**Figure 4:**
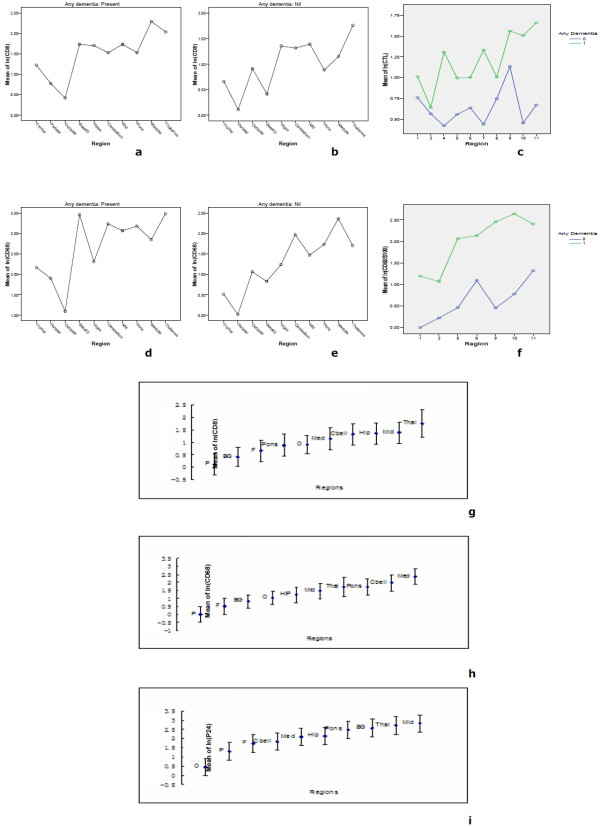
**Similarity of distributional trends of CD8, CD68 and P24 in the brain**. Linear models were used to assess the effect of region within patients. We observed that the deeper midline and mesial temporal structures of the brain were much more involved than other CNS regions as evidenced by CD8 infiltration (a), CD68 infiltration (d) along with P24 antigen (i), respectively, in HAD patients. In contrast, in HIV non-dementia patients, very similar and comparable distributional trend was found at the level of CD8 (b) and CD68 (e) infiltration patterns, although there was complete absence of P24 antigen staining across all the regions. When the data of CD8 and CD68 of HAD and HIV non-dementia patients were combined (g and h), not surprisingly the trends are very similar with P24 distributional trend in HAD patients. The CTL and activated macrophage results confirm that trend again (c and f).

We have employed linear models to assess the regional influence within patients with and without dementia, which treats patient ID as a random effect and region as a fixed effect and these were fitted into the log (CD8), log(CD68), log (P24) transformation data. Notably, in both patients with dementia, the deeper midline and mesial temporal structures of the brain (basal ganglia, mid brain, pons, medulla, thalamus, and hippocampus, and cerebellum) showed frequent and extensive P24 antigen positive cells (Fig.3) in comparison to other lobes and sub-regions of the lobes, where diminishing prevalence of P24-positivity was observed. Coincident with these findings was a parallel distributional trend of CD8+ T cells and CD68+ macrophages among all the regions studied from these two HAD patients (Fig.4a and 4d). This was also consistent with our pathological findings for the two patients. Thus, the most unique observation of our studies is that although parallel distributional trend of cellular infiltration of CD8+ T cells and CD68+ macrophages was seen in both HIV non-dementia and HAD patients (Fig.4a and 4d, b and 4e), the trend of cellular infiltration of CD8+ T cells and CD68+ macrophages following P24 staining was unique to HAD patients. Further, our linear model supported this unique distributional trend of P24, CD8+ T cells and CD68+ macrophages in HAD patients with strict predilection to the deeper midline and mesial temporal structures (p < 0.05), which appeared to be highly affected by selective HIV-related pathology and viral-mediated damage of the brain tissue (Fig.4i). In order to compare the distribution of P24 and cellular infiltration among all our patients, we combined HAD and HIV non-dementia data, which further confirmed the validity of our observations (Fig. 4g, h and 4i). Overall, the complete absence of P24 in all the brain regions of HIV non-dementia was the most discernible difference between patients with and without dementia.

#### Activation of macrophage and CTL in deeper midline and mesial temporal structures of the CNS

**Figure 5:**
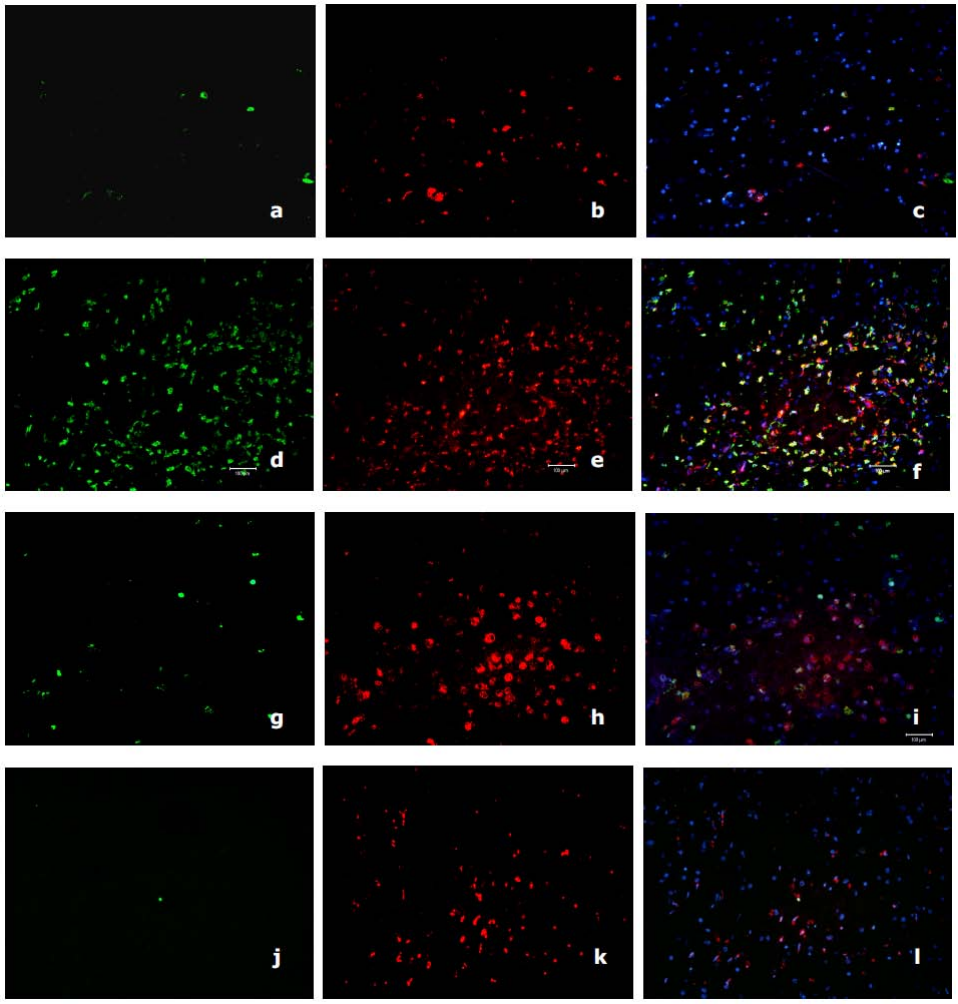
**Immunohisto-fluorescent S100/CD68 double staining of HAD and HIV non-dementia patient**. Immunohisto-fluorescent S100(green)/CD68(red) double staining of HAD (patient A parietal lobe: a, b and c; patient A medulla: d, e and f) and HIV non-dementia (patient D medulla: g, h and i; patient F medulla: j, k and l) patient. These data comply with our immuno-histochemistry staining in the deeper midline and mesial temporal structures of the brain. The CD68 staining was much more pronounced than lobes and there are comparable levels of CD68 staining, while the S100 stained cells are significantly less in HIV non-dementia patients.

**Figure 6:**
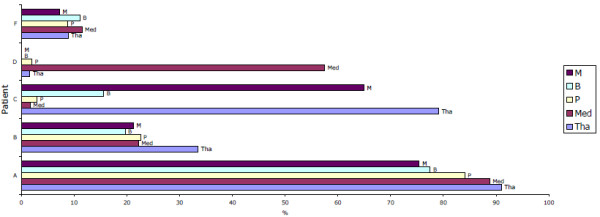
**Percentage of activated macrophages in the deeper midline and mesial temporal structures of the brain**. Percentage of activated macrophages in the deeper midline and mesial temporal structures of the brain, based on the ratio of S100A8+CD68+ cells count to CD68+ cell counts in each of the CNS regions shown. In general, high activation rate was observed in HAD patients, except the mid brain and thalamus in the patient C and medulla in patient D. M: mid brain, B: basal ganglia, P: pons, MED: medulla, Tha: thalamus.

Anti-S100 A8 antibody was used in conjunction with the anti-CD68 antibody to identify activated macrophages [34-36]. Interestingly, the activated macrophages, dually stained by S100 A8 and CD68, were mostly observed in the deeper midline and mesial temporal structures of the brain, especially in medulla, pons and midbrain regions while only to a lesser degree in the frontal and parietal lobes (Fig.5). This regional distribution of activated macrophages (Fig.4f) was consistent with the distribution of HIV infection and intense infiltration of macrophages, especially in the deeper midline and mesial temporal structures of the brain. In addition, the numbers of activated macrophages in HAD patients were 4.03 fold higher than HIV non-dementia patients (p < 0.01) and the percentage of activated macrophages accounted for up to 91% of total macrophages in dementia patients, which was higher than in HIV non-dementia patients in most regions (Fig.6). Overall, these results strongly support that activated macrophages alone are not enough to trigger HAD. In fact, together the productive HIV infection of macrophages and their localization in the deeper midline and mesial temporal structures of the brain are vital for neurologic manifestation of HIV disease.

Further, we used anti-granzyme B antibody to identify CD8+ CTL. Our results confirmed the distribution trend observed in previous result section, which is the involvement of deeper midline and mesial temporal structures of the CNS (Fig.4c). However, the variation between 2 HAD patients was bigger than we expected. The CTL in the patient A in every region was significantly higher than all 4 HIV non-dementia patients. Whereas, patient B showed only limited CTL, which was comparable to HIV non-dementia patients. Interestingly, statistically significant difference (p < 0.01) was found when we compared patients A and B as a group against all 4 HIV non-dementia patients.

#### Unique nature of sequences derived from Laser-captured macrophages from the middle part of the brain

**Figure 7:**
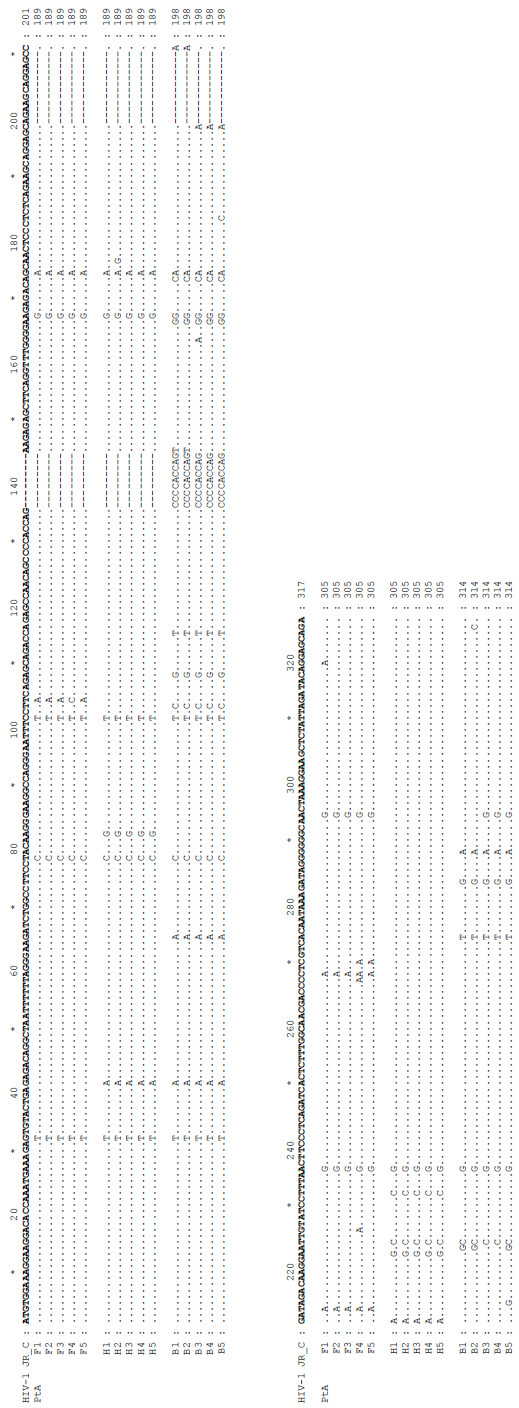
**DNA sequence alignment of the HIV GAG-POL for different regions of patient A**. DNA sequence alignment of HIV GAG-POL (325 bp region) for different regions of patient A showing nucleotide changes common and unique to each region. F: frontal, H: hippocampus, B: basal ganglia

**Figure 8:**
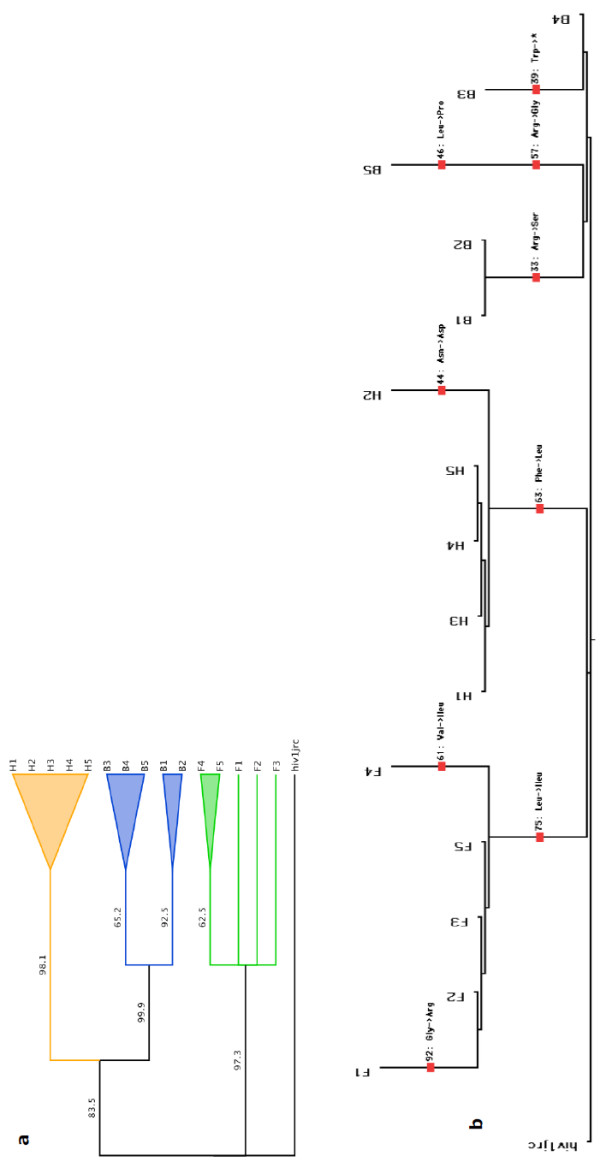
**Phylogenetic analysis and splits tree of the HIV GAG-POL for different brain regions of patient A**. (**a**). Phylogenetic analysis based on the nucleotide sequence of the 325 bp region of HIV-1 GAG-POL from patient A. HIV-1 JRCSF was used as an outlier. (**b**). Splits tree phylogenetic reconstruction based on the protein sequence of the pol region. As the 325 bp nucleotide sequence contained 2 separate overlapping coding regions of the Gag and Pol genes, we excised the first 45 nucleotide bases from the alignment for translating the Pol section of the gene to amino acids for phylogenetic reconstructions. The tree shows inter-phylogenetic relationships based on single amino acid changes (shown for each region and between regions at appropriate branches along with positions). F: frontal, H: hippocampus, B: basal ganglia.

Since HIV-infected macrophages appear to play a vital role in HAD, multiple clones of HIV provirus (325 bp gag gene fragment) were analyzed from catapulted macrophages positive for P24 antigen. It showed a large deletion (CCCCACCAG + T) in two sequences between positions 134-143, which was unique to the basal ganglia (Fig.7). Similarly, distance-based calculation and 99% bootstrap estimations further confirmed the uniqueness of hypothalamus-derived sequences H1, H3, H4 and H5. Both Neighbor joining and Parsimony phylogenetic estimations at the nucleotide level identified distinct grouping of sequences from the respective brain regions, suggesting independent evolution of virodemes (Fig.8a). Since the 325 bp gag region analyzed in this study is an overlap between gag and pol genes, we further analyzed the pol gene amino acid sequences. Amino acid changes supporting region specificity were a Leu to a Ileu at amino acid position 75, which were unique to F1-F5 sequences; and Phe to Leu change at position 63, which grouped H1-H5 sequences from others. Other region-specific changes can be visualized in Fig.8b.

## Discussion

In this study, we have investigated cellular infiltration of macrophages and CD8+ T cells and their activation states in diverse areas of the brain, especially in concomitance with HIV in order to define regional predilection of these cell types and their possible association with neurologic manifestation of HIV disease or HAD. Here, we provide the first evidence showing **1**. That macrophage and CD8+ T cell infiltration and their activation alone was not enough for the development of dementia. **2**. That the topographic patterns of macrophage/CD8+T cell distribution and levels of activation in diverse areas the CNS were comparable between patients with and without dementia. **3**. That the major distinction between HAD and HIV non-dementia patients was the predilection of HIV-infected macrophages and CD8+ T cells to the deeper midline and mesial temporal structures (thalamus, medulla, hippocampus, cerebellum, basal ganglia and pons) uniquely in HAD patients. This further correlated with HIV-related pathology in these parts of the CNS and also with the neurological manifestation of HIV disease in both HAD patients. Further, this distribution pattern was also consistent with the activation patterns, confirmed by S-100A8 immunohistochemical staining, which was also pronounced in the deeper midline and mesial temporal structures. Thus the preponderance of activated/infected CD8+ T cells and CD68+ macrophages in the midline and mesial temporal structures of the brain is the feature unique to HAD patients. In contrast, even though considerable macrophage and CD8 infiltration was observed in diverse areas of the brain of HIV non-dementia patients, there was complete lack of P24 antigen staining and low level of cellular activation, which indicates either the absence or lower than detectable level of HIV in the CNS of HIV non-dementia patients.

It is known that HIV in the periphery and the CSF may impair the integrity of the blood-brain barrier (BBB), which makes the BBB permeable to cellular infiltrates or the recruitment of immune cells into the CNS [37-39]. However, the strength of the immune system and the quality of immune cells migrating to the CNS determine the onset of neurological manifestation of HIV disease. Thus, the immuno-suppression of the host by HIV may have strong bearing on the appearance of productively infected macrophages, which was the critical feature observed in both HAD patients examined in our study. The actual relationship, between HIV-1 proviral load and clinical diagnosis of HIV-associated dementia (HAD), remains to be established. A clear determination of neuroanatomic distribution of HIV-1 proviral loads in diverse areas of the brain (including deeper midline and mesial temporal structures) of individuals with and without HAD may facilitate elucidation of the relationship between tissue proviral load and HAD, and may also explain whether regional predilection of provirus also correlates with macrophage and CD8+ T cell infiltration and active replication of HIV in HAD patients, as we have observed. Taken together, our findings lead to the supposition that the predilection of HIV-infected macrophages and CD8+ T cells to the deeper midline and mesial temporal structures (which includes the brainstem), along with the unique architecture of proviral sequences appear to play a vital role in the development of dementia in HIV patients.

These and our recently published data [30] are consistent with previous findings showing that the infection was most frequent in the deeper midline and mesial temporal structures, whereas the choroids plexus (CPx) showed no productive infection [28,40]. Further, another semi-quantitative study by Neuen-Jacob [29], which analyzed topographical distribution of HIV core antigen in relation to macrophages showed that deep grey matter, in particular putamen and thalamus, were involved in every case, irrespective of disease stage. It is also important to reiterate that HIV infection may incur indirect modalities and our data confirm the active HIV infection of the CNS being the vital feature for neurologic manifestation. Without productive infection of macrophages in the deeper midline and mesial temporal structures by HIV, the trigger for HAD does not occur. Thus, our supposition for the localization of HIV-infected/activated macrophages and CD8+ T cells in the context of the development of dementia in HIV patients is highly relevant.

Previously, CD8+ cytotoxic T lymphocytes specific for viral antigens have been shown to accumulate and correlate with the central nervous system dysfunction in SIV or HIV infection [25,27]. However, studies on macaques by Sopper [20], have shown that increased infiltration of CD8+ cytotoxic T ymphocytes specific for viral antigens were detected only in the CSF of slow progressors. Consistently, we observed no differences at the level of CD8+ T cell infiltration and infection in two HAD patients analyzed in our study, but much higher CTL in the patient who died within 6 years, and very little in the patient who died within 40 weeks. Thus, our data suggest that slow progressors may display strong intrathecal immune response suggesting a possible protective role or delay of the virus-specific immunity in HIV-induced central nervous system disease, as opposed to rapidly progressing HAD patients.

Overall, why deeper parts appear to be so much more involved rather than the cortex proximal to the meningeal or ventricle area? And why the whole midline and mesial temporal parts of the brain are so severely affected histopathologically? Specific tissue vulnerability may explain part of the puzzle, but not the underlying reasons regarding the specific topographic distribution of HIV and cellular infiltrates in HAD patients. It is known that the brainstem disturbance can be associated with dementia through interference with arousal systems. Pathologically, gross examination of the brain in progressive supranuclear palsy (PSP) shows midbrain atrophy. Further, in PSP neuronal loss and neurofibrillary tangles in the basal ganglia, diencephalon and brainstem have been observed. The substantia nigra, subthalamic nucleus and pontine base are typically involved, as well as the ventral anterior and lateral thalamic nuclei. The cerebellar dentate nucleus may show degeneration [41]. In addition, in the context of HIV basal ganglia hyperatrophy has been shown to often accompany mild cognitive impairment [42]. Thus, taken together, it leads us to postulate that the productive infection of HIV in the midline and mesial temporal structures is crucial to the occurrence of dementia in HIV patients. We wish to reiterate that we do not imply that dementia can exclusively be caused by brainstem disease -more that it can exacerbate probably through several mechanisms - diminished arousal and therefore attention.

## Conclusion

Strong predilection of infected macrophages and CD8+ T cells was typical of the deeper midline and mesial temporal structures uniquely in HAD patients, which has some influence on neurocognitive impairment during HIV infection.

## Abbreviations

Abbreviations used in this paper: HAART: highly active antiretroviral therapy; HAD: HIV-associated dementia; ln: Log;

## Competing interests

The authors declare that they have no competing interests.

## Authors' contributions

LZ fully performed the work, analyzed data and wrote the paper. RR, KH and CG performed the work on HIV activation using S-100, VV contributed to immunohistochemical analysis, TN extensively contributed to neurologic and immunohistochemical results interpretation and analysis, YSH contributed to phylogenetic analysis, NKS provided ideas, designed the research project, planned and supervised this work and contributed to the writing.

## Pre-publication history

The pre-publication history for this paper can be accessed here:

http://www.biomedcentral.com/1471-2334/9/192/prepub

## Supplementary Material

Additional file 1 **Negative and positive controls for CD8, CD68 and P24 staining**. Negative and positive controls for CD8, CD68 and P24 staining. Tonsil tissue from patients with tonsillitis was used as CD8 and CD68 positive controls, and the same tissue was used as negative controls by omitting the primary antibodies. HIV positive tonsil tissue was used as P24 positive controls while the same tissue was used as negative controls by omitting the P24 antibody. Click here for file

Additional file 2 **Immunohistochemistry P24/CD8 and P24/CD68 double staining of HIV non-dementia patients**. Immunohistochemistry P24(brown)/CD8(red) and P24(brown)/CD68(red) double staining of HIV non-dementia patients (patient C, patient D and patient F) in hippocampus (a and b), cerebellum (c and d), mid brain (e and f), and pons (g and h) regions. Comparable intensity of CD68 (b, d, f and h) and noticeable CD8 (a, c, e and g) staining were observed in HIV non-dementia patients, but these cells were negative for P24 antigen in all the brain regions studied for all 4 HIV non-dementia patients. Click here for file
