# Supplementary figures and images for: Evidence for predilection of macrophage infiltration patterns in the deeper midline and mesial temporal structures of the brain uniquely in patients with HIV-associated dementia
Source: BMC Infect Dis. 2009 Dec 2;9:192. doi: 10.1186/1471-2334-9-192 (PMC2792226; doi:10.1186/1471-2334-9-192)

**Negative Control**

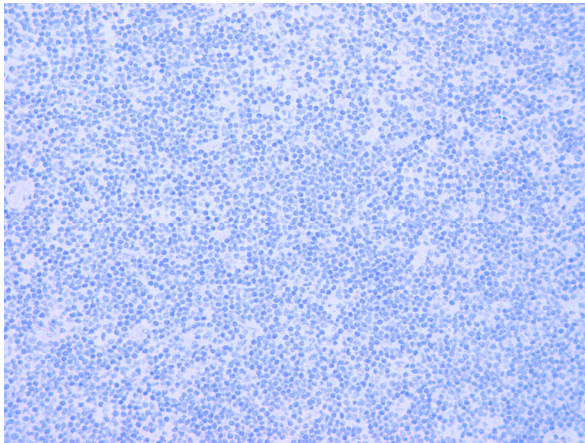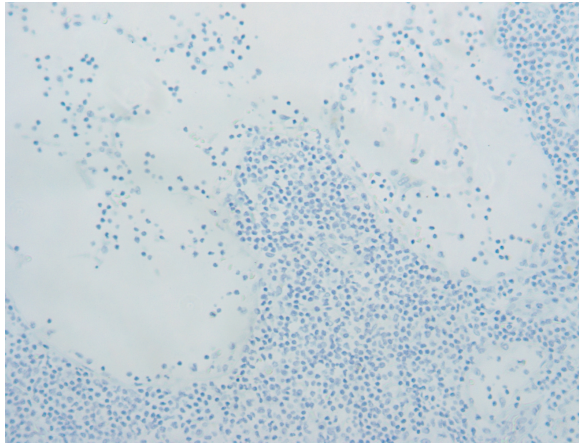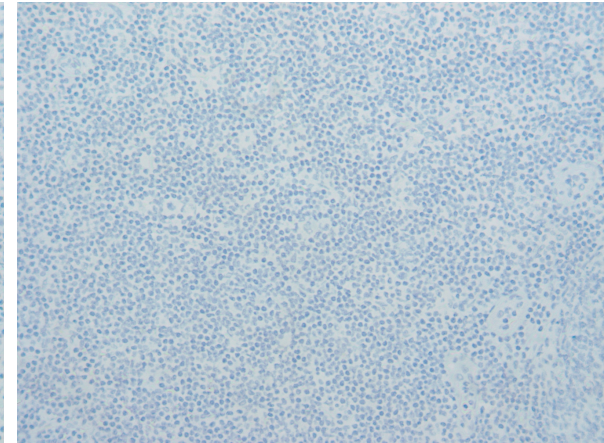

**CD8**

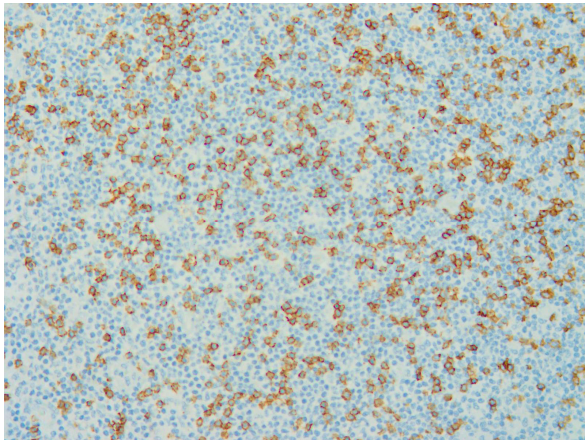

**CD68**

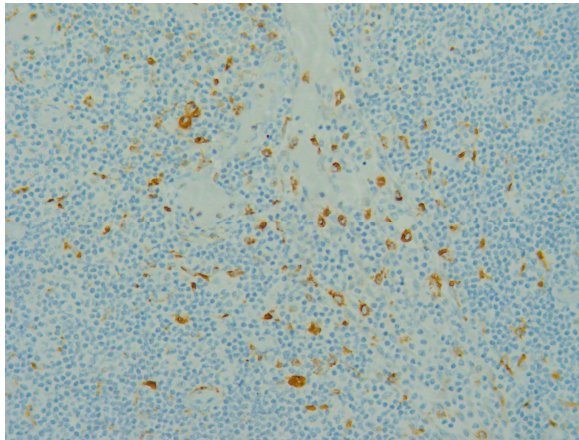

**P24**

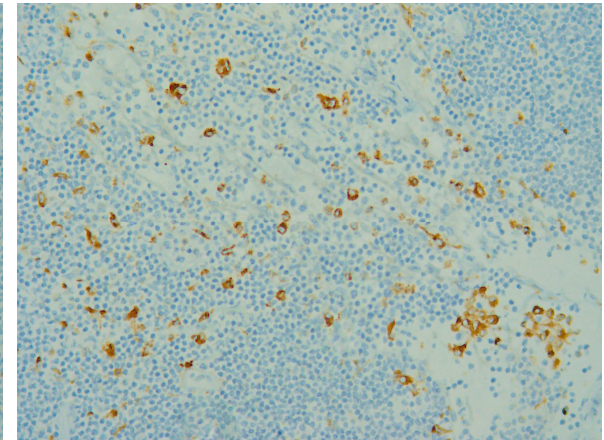

**Positive Control**

Supplement: Additional file 1 — Negative and positive controls for CD8, CD68 and P24 staining. Negative and positive controls for CD8, CD68 and P24 staining. Tonsil tissue from patients with tonsillitis was used as CD8 and CD68 positive controls, and the same tissue was used as negative controls by omitting the primary antibodies. HIV positive tonsil tissue was used as P24 positive controls while the same tissue was used as negative controls by omitting the P24 antibody. [file 1471-2334-9-192-S1.PDF]

**P24/CD8**

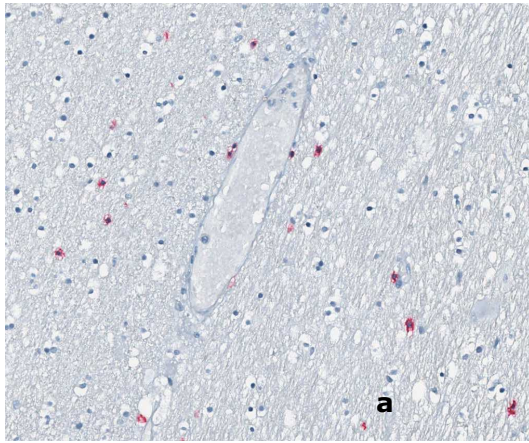

**P24/CD68**

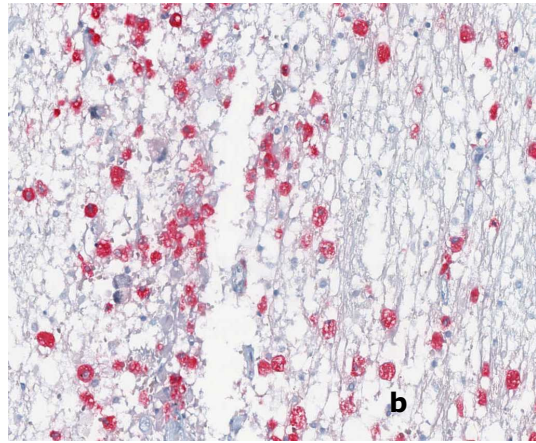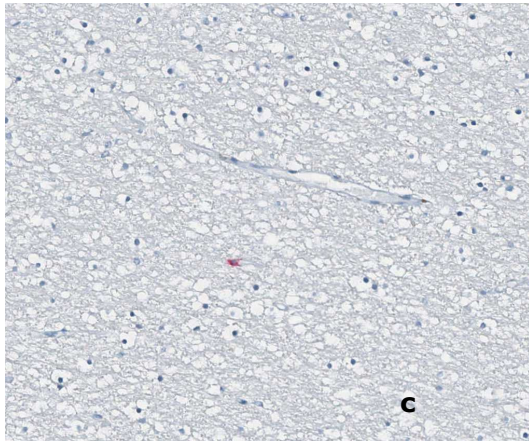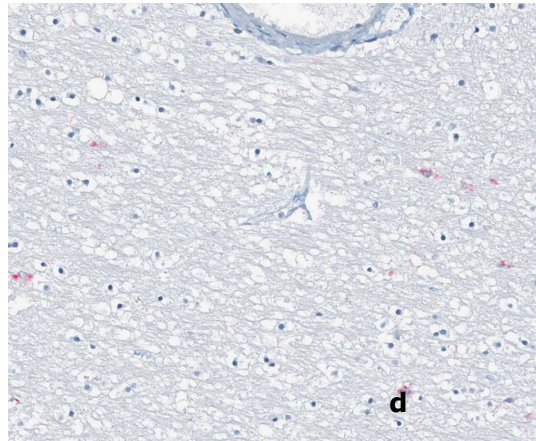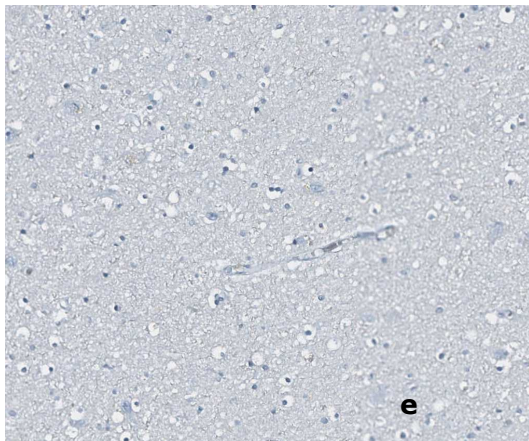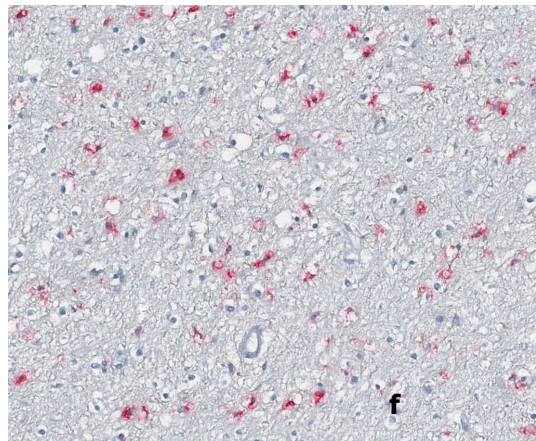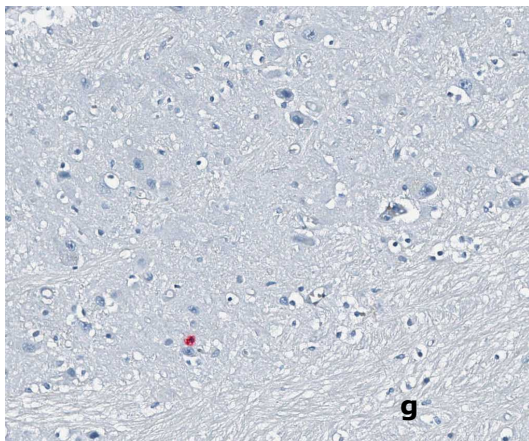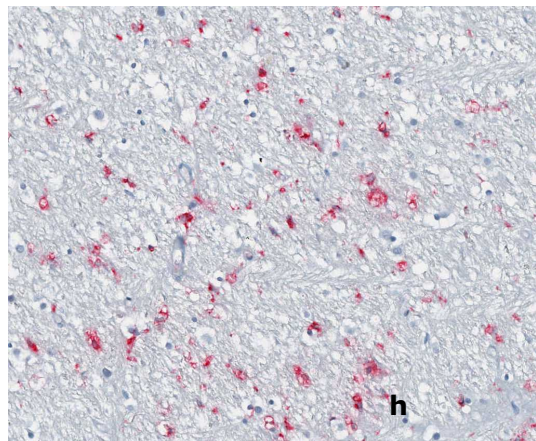

**Patient C**

**P24/CD8**

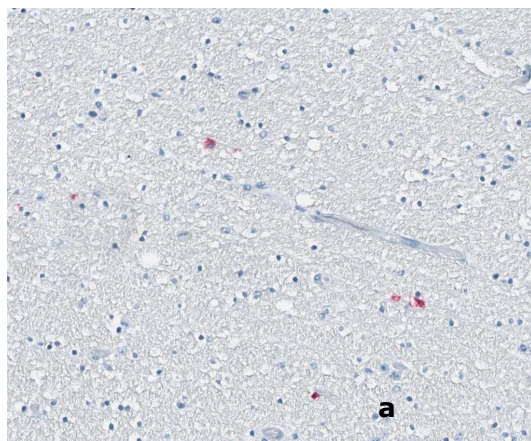

**P24/CD68**

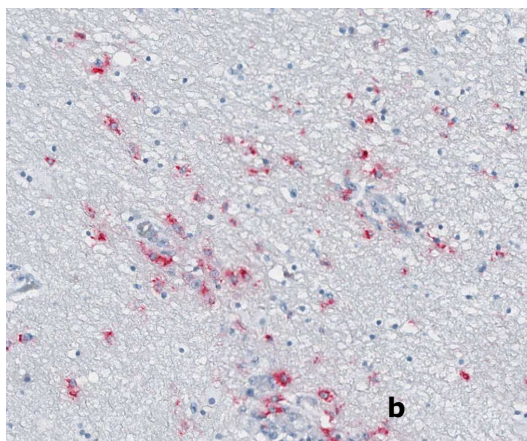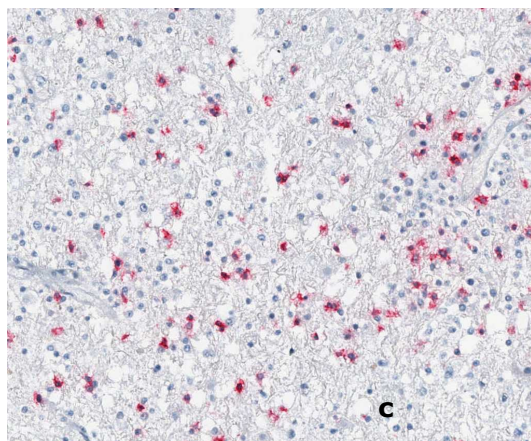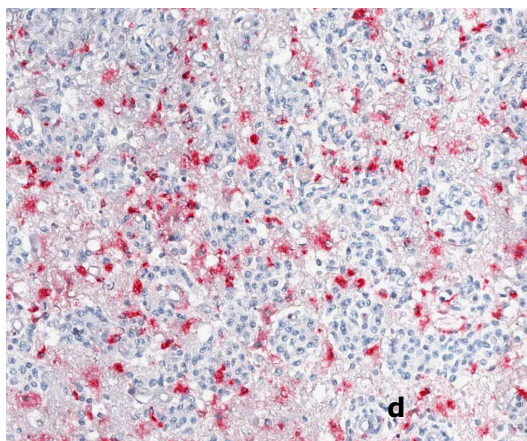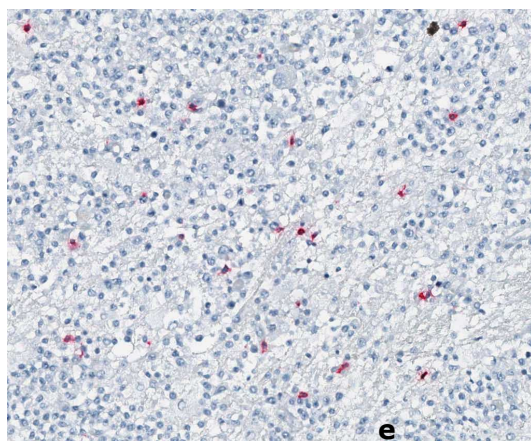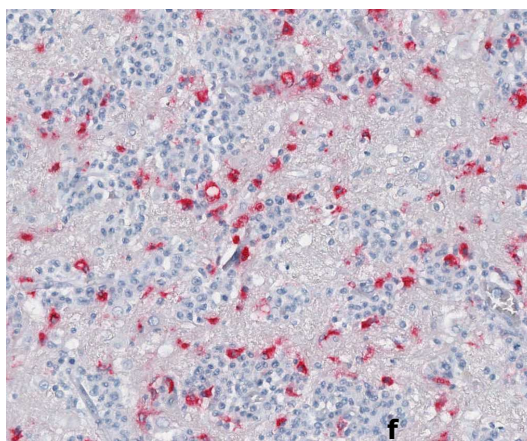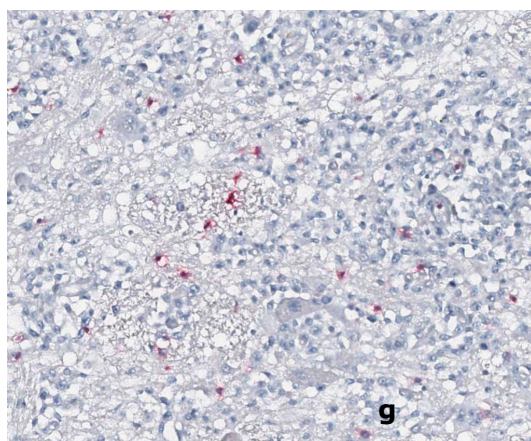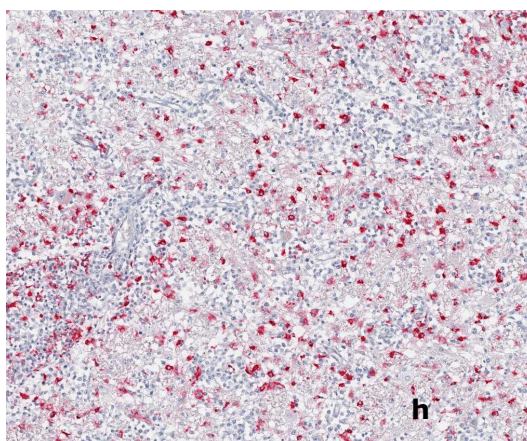

**Patient D**

**P24/CD8**

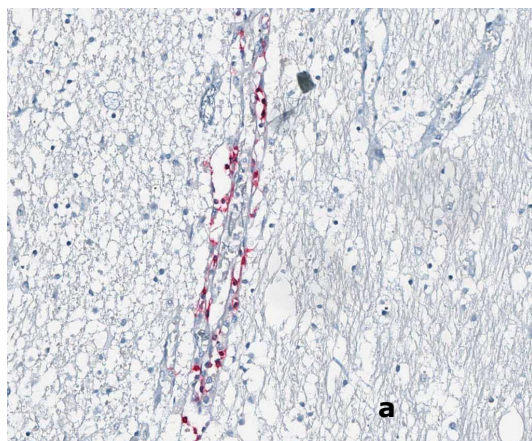

**P24/CD68**

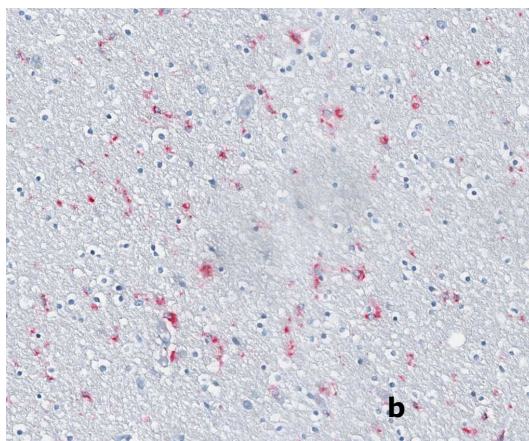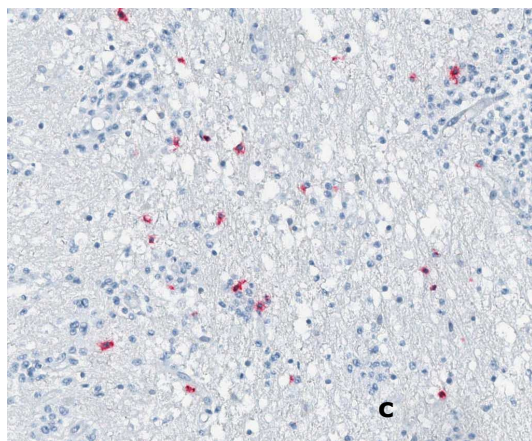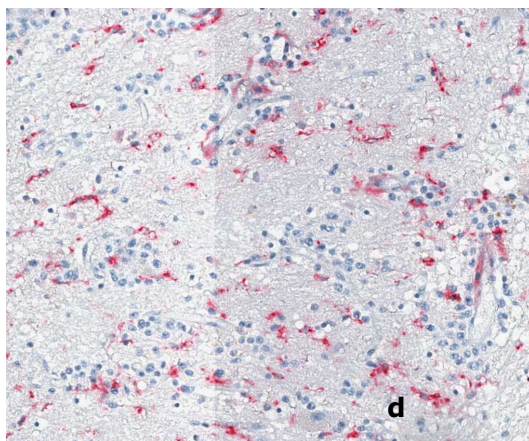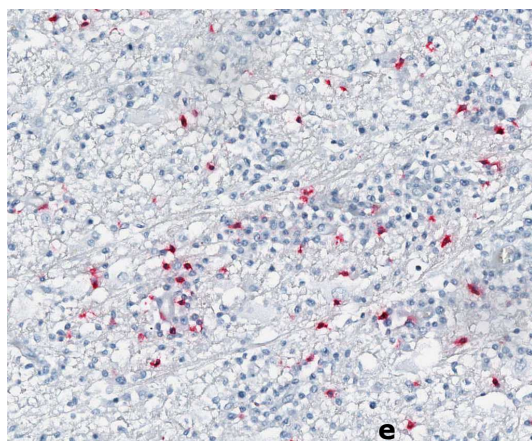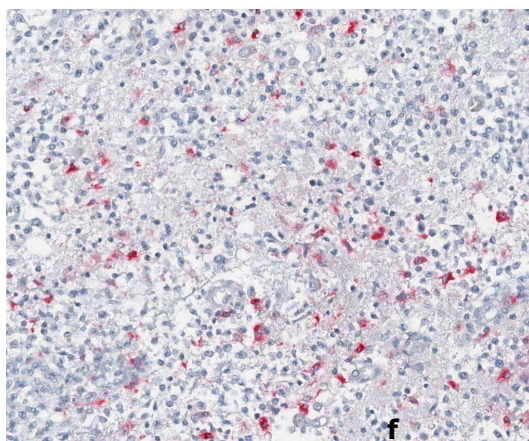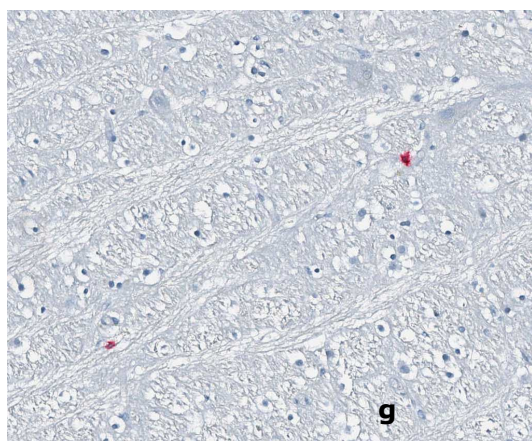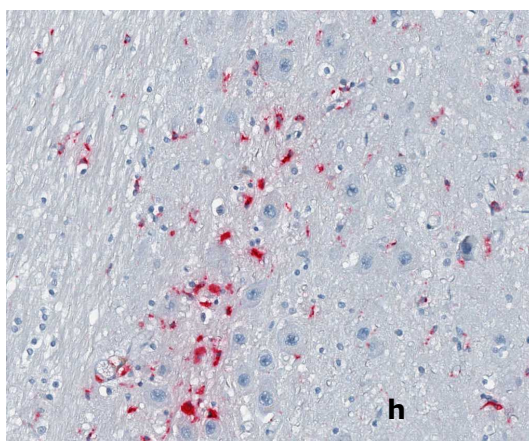

**Patient F**

Supplement: Additional file 2 — Immunohistochemistry P24/CD8 and P24/CD68 double staining of HIV non-dementia patients. Immunohistochemistry P24(brown)/CD8(red) and P24(brown)/CD68(red) double staining of HIV non-dementia patients (patient C, patient D and patient F) in hippocampus (a and b), cerebellum (c and d), mid brain (e and f), and pons (g and h) regions. Comparable intensity of CD68 (b, d, f and h) and noticeable CD8 (a, c, e and g) staining were observed in HIV non-dementia patients, but these cells were negative for P24 antigen in all the brain regions studied for all 4 HIV non-dementia patients. [file 1471-2334-9-192-S2.PDF]
